# Performance of the Intermittently Scanned Continuous Glucose Monitoring (isCGM) System during a High Oral Glucose Challenge in Adults with Type 1 Diabetes—A Prospective Secondary Outcome Analysis

**DOI:** 10.3390/bios11010022

**Published:** 2021-01-15

**Authors:** Othmar Moser, Norbert Tripolt, Peter Pferschy, Anna Obermayer, Harald Kojzar, Alexander Mueller, Hakan Yildirim, Caren Sourij, Max Eckstein, Harald Sourij

**Affiliations:** 1 Cardiovascular Diabetology Research Group, Division of Endocrinology and Diabetology, Department of Internal Medicine, Medical University of Graz, 8036 Graz, Austria; norbert.tripolt@medunigraz.at (N.T.); peter.pferschy@medunigraz.at (P.P.); a.obermayer@medunigraz.at (A.O.); Harald.Kojzar@medunigraz.at (H.K.); Alexander.Mueller@medunigraz.at (A.M.); hakan.yildirim@stud.medunigraz.at (H.Y.); caren.sourij@medunigraz.at (C.S.); Max.Eckstein@uni-bayreuth.de (M.E.); ha.sourij@medunigraz.at (H.S.); 2 Division of Exercise Physiology and Metabolism, Department of Sport Science, University of Bayreuth, 95445 Bayreuth, Germany; 3 Center for Biomarker Research in Medicine (CBMed), 8010 Graz, Austria; 4 Exercise Physiology, Training & Training Therapy Research Group, Institute of Sports Science, University of Graz, 8010 Graz, Austria; 5 Zayed Center for Health Sciences (ZCHS), United Arab Emirates University, Al Ain 15551, UAE

**Keywords:** isCGM, glycemia, accuracy

## Abstract

To assess intermittently scanned continuous glucose monitoring (isCGM) performance for different rates of change in plasma glucose (RCPG) during glycemic challenges in type 1 diabetes (T1D). Nineteen people with T1D (7 females; age 35 ± 11 years; HbA_1c_ 7.3 ± 0.6% (56 ± 7 mmol/mol)) performing two glycemic challenges (OGTT) were included. During OGTTs, plasma glucose was compared against sensor glucose for timepoints 0 min (pre-OGTT), +15 min, +30 min, +60 min, +120 min, +180 min, and +240 min by means of median absolute (relative) difference (MARD and MAD) and Clarke Error Grid (CEG), then was stratified for RCPG and glycemic ranges. Overall, MARD was 8.3% (4.0–14.8) during hypoglycemia level 1 18.8% (15.8–22.0), euglycemia 9.5% (4.3–15.1), hyperglycemia level 1 9.4% (4.0–17.2), and hyperglycemia level 2 7.1% (3.3–11.9). The MARD was associated with the RCPG (*p* < 0.0001), detailing significant differences in comparison of low, moderate, high, and very high RCPG (*p* = 0.014). Overall, CEG resulted in 88% (212 values) of comparison points in zone A, 12% (29 values) in zone B, and 0.4% (1 value) in zone D. The isCGM system was accurate during OGTTs. Its performance was dependent on the RCPG and showed an overestimation of the actual reference glucose during hypoglycemia.

## 1. Introduction

Intermittently scanned continuous glucose monitoring (isCGM) systems display interstitial glucose levels at the time when a scan with a reader device is performed. isCGM reduces the time spent in hypoglycemia (<70 mg/dL; 3.9 mmol/L) and improves long-term glycemic control as assessed by glycated hemoglobin (HbA_1c_) (mean of glycemia over the last 2–3 months) [1]. The use of isCGM was also associated with improved quality of life and less anxiety when compared against regular blood glucose measurements from fingertip (SMBG) [2]. Intriguingly, higher rates of sensor glucose scanning were associated with improvements in glycemia, expressed by increased time spent in range (near normal physiological glucose concentration; 70–180 mg/dL (3.9–10.0 mmol/L)) and reduced time spent above this range (>180 mg/dL (>10.0 mmol/L)) [3]. Taking into account that people with diabetes perform around 16 scans per day [3], and therapy decisions (exogenous insulin administration and carbohydrates intake) of particular people with type 1 diabetes (T1D) are based on the sensor glucose levels accompanied by trend arrows that indicate the rate of change in glucose levels, there is a need to investigate isCGM under different glycemic challenges [4]. For example, as shown for physical exercise, the accuracy of the isCGM system decreased significantly [5,6,7], and the device accuracy was further reduced during hypoglycemia (<70 mg/dL; 3.9 mmol/L) [8], where accurate values are needed to avoid severe hypoglycemia. As shown in a retrospective analysis of two continuous glucose monitoring (CGM) systems, accuracy of sensor glucose levels was affected by rapidly changing blood glucose concentrations [9]. Thus, if blood glucose concentrations changed more than 3 mg/dL/min, the median absolute relative difference (MARD) between the sensor glucose and the actual blood glucose was ~28% [9]. Considering that isCGM is getting more widely used, replacing SMBG, there is the necessity to thoroughly assess isCGM performance in relation to the rate of change in plasma glucose (RCPG). The aim of the analysis was to investigate the accuracy of isCGM systems depending on the RCPG in a setting of rapidly changing glucose values, namely oral glucose tolerance tests (OGTT), in people with T1D. 

## 2. Materials and Methods 

This was a single-center, prospective secondary outcome analysis performed in adults with T1D. The study protocol was approved by the local ethics committee (30–238 ex 17/18) and registered at the German Clinical Trials Register (drks.de; DRKS00016148). The study was conducted in conformity with the declaration of Helsinki and Good Clinical Practice. Before any trial related activities, potential participants were informed about the study protocol, and participants gave their written informed consent. Sample size estimation was based on the primary outcome of the study that was defined as the assessment of the impact of prolonged fasting (36 h vs. 12 h) on glycemic parameters within the OGTT. 

### 2.1. Eligibility Criteria

Eligibility criteria included a diagnosis of T1D > 12 months, age > 18 years, treatment with exogenous insulin via multiple daily injections (MDI) or continuous subcutaneous insulin infusion (CSII), c-peptide level ≤ 0.3 nmol/L, HbA_1c_ < 9.5% (<80 mmol/mol), a body mass index (BMI) 20–29.9 kg/m^2^, and no diabetic ketoacidosis or severe hypoglycemia requiring external assistance within the last 12 months. Additionally, participants had to use an isCGM (FreeStyle Libre 1, Abbott, IL, USA) for glucose monitoring. 

### 2.2. Screening Visit

At the screening visit, eligibility criteria were assessed. For the assessment of diabetes specific parameters and general health, a venous blood sample was drawn, and resting electrocardiogram and blood pressure measurements were performed. Participants were told to ensure that, in the case of a potential sensor expiration at the time of an OGTT, the sensor was changed at least 24 h prior to the start of the OGTT. The isCGM sensor was placed in line with instruction leaflet at the back of the upper arm.

### 2.3. Trial Visits

Based on the primary outcome of the study protocol, participants were enrolled in two non-randomized trial visits: an OGTT after 12 h of fasting and after 36 h of fasting. During the fasting period, participants were allowed to consume water and consume carbohydrates in case of hypoglycemia. If two or more hypoglycemic events occurred, the visit was rescheduled. 

For both OGTTs, 75 g of glucose (oligosaccharide) were dissolved in 300 mL water and consumed accompanied by the same individual dose of bolus insulin. The individual bolus insulin dose was based on the participants’ regular carbohydrate to bolus insulin ratio for breakfast, as assessed at the screening visit. Venous blood samples were drawn for the assessment of reference plasma glucose (hexokinase method, Cobas 8000, Hoffmann-La Roche, SUI), and a scan with the reader device was performed (interstitial glucose) at the following time points: 0 min (pre-OGTT), +15 min, +30 min, +60 min, +120 min, +180 min, and +240 min. At these time points, sensor glucose values were compared to the accompanied plasma glucose value. Capillary blood glucose measurements were performed for safety reasons in case of fast decreasing or increasing sensor glucose levels. OGTTs were discontinued early if the capillary blood glucose concentration dropped below 3.9 mmol/L (70 mg/dL) and, immediately, 15–30 gr carbohydrates were orally ingested by the participants.

### 2.4. Statistical Analysis

Plasma glucose was compared against sensor glucose for the same time point by means of median absolute difference (MAD), median absolute relative difference (MARD), and Clarke Error Grid (CEG) analysis for overall data. Furthermore, these analyses were performed for data stratified based on glycemic ranges and RCPG. Glycemic ranges were defined as hypoglycemia level 1 (<70 mg/dL (<3.9 mmol/L)), euglycemia (70–180 mg/dL (3.9–10.0 mmol/L)), hyperglycemia level 1 (181–250 mg/dL (10.1–13.9 mmol/L)), and hyperglycemia level 2 (>250 mg/dL) (>13.9 mmol/L)) [10]. RCPG was divided as follows: low RCPG from minimum—25th percentile; moderate RCPG 26th percentile—median; high RCPG median—75th percentile; very high RCPG above the 75th percentile. Hypoglycemia level 2 (<54 mg/dL (<3.0 mmol/L)) was not assessed, since no levels occurred for this range. The following data were assessed for distribution by Shapiro Wilk normality test: subgroups of MARD including glycemic range and RCPG groups. Comparison of MARD for subgroups of glycemia (hypoglycemia levels 1 vs. euglycemia vs. hyperglycemia levels 1 and 2) and subgroups of RCPG (low vs. moderate vs. high vs. very high RCPG) were analyzed by means of Kruskal–Wallis test with post-hoc Dunn’s testing and were further assessed by linear regression modeling (*p* < 0.05). For these assessments, we hypothesized significant differences for MARDs based on glycemia (highest MARD for hypoglycemia level 1) and based on RCPG (highest MARD for very high RCPG). Statistics were performed with GraphPad Prism Version 8.0.2 (GraphPad Software, Inc., San Diego, CA, USA) and IBM SPSS Statistics Version 26 (IBM, New York City, NY, USA).

## 3. Results

Nineteen people with T1D (7 females; age 35 ± 11 years; body mass index (BMI): 24.5 ± 2.7 kg/m^2^; HbA_1c_ 7.3 ± 0.6% (56 ± 7 mmol/mol), diabetes duration 18 ± 11 years; 12 MDI/7 CSII; total daily insulin dose 41 ± 15 IU) were included in this analysis. Out of 266 potential points of comparison, 242 were available. In six visits, hypoglycemia occurred, hence the last points of comparison or more were not available for analyses. In nine cases, plasma glucose assessment failed, and one participant did not perform the second trial visit. isCGM sensor glucose never failed to display a value (100%). Participants injected a mean ± SD bolus insulin dose of 5.6 ± 1.7 IU prior to the start of each OGTT. 

### 3.1. Median Absolute Relative Difference (MARD)

All data were not normally distributed. MARD and MAD for the different glycemic ranges and RCPG are given in Table 1. MARD was significantly different in comparison to the three RCPG groups (*p* = 0.014), following a linear regression equation for ARD of y = 0.026*x + 0.068 (r^2^ = 0.12, *p* < 0.0001). The MARD based on the difference glycemic ranges was also found to be significantly different (*p* = 0.026).

### 3.2. Clarke Error Grid (CEG) Analysis

**Figure 1:**
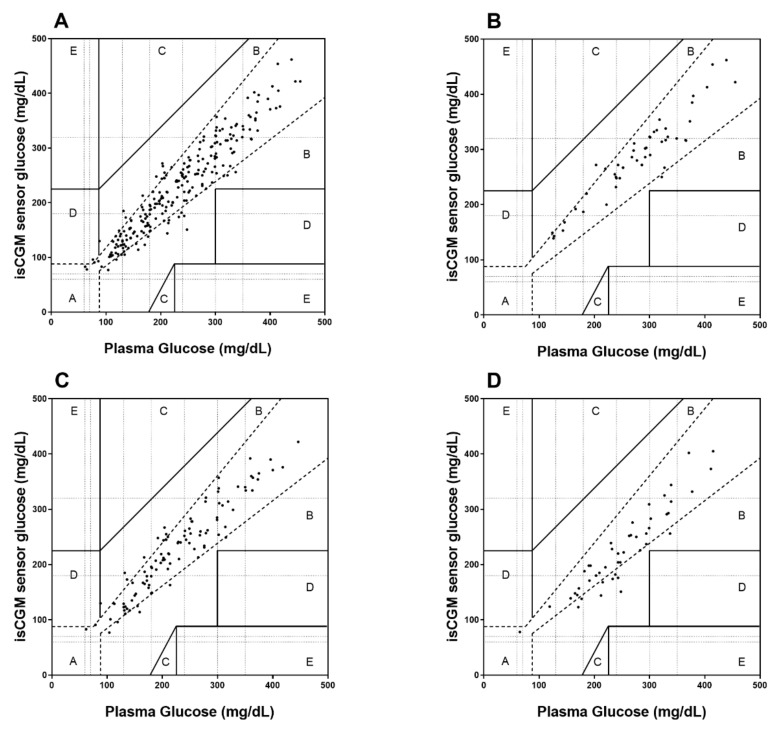
Clarke Error Grid (CEG) analysis for the assessment of isCGM accuracy for overall data (**A**), during low RCPG (**B**), moderate RCPG (**C**), and high RCPG (**D**) with respect to clinical decisions.

Overall, CEG resulted in 88% (212 values) of comparison points in zone A (no effect on clinical action), 12% (29 values) in zone B (altered clinical action with small or no significant effect on clinical out-come), and 0.4% (1 value) in zone D (altered clinical action that could have significant medical risk) (Figure 1). During low RCPG, 92% (47 values) of comparison points were located in zone A and 8% (4 values) in zone B, during moderate RCPG, 86% (89 values) in zone A and 14% (14 values) in zone B, and during high RCPG, 83% (45 values) in zone A and 17% (9 values) in zone B. No values were located in zones C (altered clinical action with the probability of affecting clinical outcome) and E (altered clinical action that could have dangerous consequences). 

## 4. Discussion

This is the first study that assessed the performance of isCGM during a standardized oral glucose challenge in adults with T1D. In general, the MARD for the isCGM system compared with reference plasma glucose values was found at 8.3% during the OGTTs, which is more accurate in comparison to previous studies when assessed during carbohydrate rich meals [11], induction of hypo- and hyperglycemia [12], 14 days of outpatient habitual life [13], and under routine environmental conditions [14]. Boscari et al. [11] observed a MARD of 14.9% for isCGM following carbohydrate-rich meals accompanied with delayed and increased bolus insulin doses. Although, in their study, glycemic challenges were performed by a regular breakfast that might result in lower rates of glucose change due to increased fat absorption, the MARD in our study was ~6% lower. In a similar study design, in which rapid glucose changes were induced during a clinical research facility phase, the MARD was found at 13.6% [12]. In an assessment of isCGM performance during habitual life conditions with at least two comparative capillary blood glucose measuring per day (and during dysglycemia), isCGM showed a MARD of 16.6 ± 11.6%. However, the sample size of this study was rather small (*n* = 8), which might have influenced their findings. When isCGM performance was evaluated while simulating routing environmental conditions including exercise and dysglycemia, the MARD was found at 13.2 ± 10.9% [14]. Even in their study, the MARD was higher than that found in our results; it can be speculated that this inaccuracy can be contributed to the exercise sessions, where it was shown that isCGM is less accurate [4].

Considering the low MARD found in our study, it can be concluded that isCGM tracks post-prandial glucose excursions accurately in situations of nutritionally induced rapid glucose changes. Although performed in people with type 2 diabetes, similar findings were found during standard meal tests, detailing that 100% of isCGM comparison points were falling within zones A and B of the Clarke and Parkes error grid analysis accompanied by a MARD of 10.7% [15]. However, in this study, it was also found that, during rapidly changing glucose concentrations, the MARD increased to 19.0%, which exceeds the number observed in our (very) high RCPG. 

In line with the low MARD, the assessment of safety for clinical decisions by means of CEG resulted in 100% of values in zones A and B for overall data. Therefore, only a small influence on therapy decisions might be observed when using sensor glucose levels for insulin administration and/or carbohydrate intake. These results are of major clinical importance, since the isCGM system is approved as a non-adjunctive device, not requiring additional blood glucose measurements from fingertip, to inject insulin and/or consume carbohydrates.

In line with previous isCGM [8,16], personal [14] as well as professional continuous glucose monitoring (CGM) performance data [17], isCGM performance deteriorated in our study during the hypoglycemic range (<70 mg/dL; 3.9 mmol/L) when compared to the eu-/ (70–180 mg/dL; 3.9–10.0 mmol/L) and the hyperglycemic range (>180 mg/dL; 10.0 mmol/L). Especially during hypoglycemia, there is a need for further improvements of isCGM and other CGM systems to ensure early and appropriate treatment against severe hypoglycemia. Furthermore, as concluded by Kovatchev [18], there is a direct association between CGM accuracy and reduced time spent in the hypoglycemia that was confirmed by clinical studies and in silico experiments. Patients’ trust in accurate sensor glucose levels might be also linked to more confident and stringent exogenous insulin adjustments, potentially improving glycemic control [18,19]. Considering these aspects of glycemic management in people living with T1D, isCGM must be accurate to divulge its full potential, even during hypoglycemia.

The main determinant for isCGM inaccuracy was the rate of change in glucose concentration, detailing a decline in accuracy for moderate to high glucose swings. As evaluated in a previous study [11], the MARD during stable glycemia (−0.5–0.5 mg/dL/min) was ~13% and deteriorated up to 17% during a rate of glucose change of 1.5 mg/dL/min. Of note, in our study, the “very high RCPG” group showed a rate of glucose change of >2.33 mg/dL/min, which is much higher than in the aforementioned study. Although we observed a similar increasing pattern of MARD with increasing RCPG in our study, it is important to note that, despite a large spectrum of rate of glucose change, MARD remained moderate and within a clinically reassuring range. Our study is not without limitations. Since only 19 participants were included in the analyses, the number of comparison points is small and additionally, it would have been of interest to compare the results of isCGM directly against other CGM systems. Furthermore, we did not record at which day within the 14 day wearing period the isCGM assessment was performed; however, as shown in a previous study, isCGM performance is stable over the 14 day period [20]. Future research is needed to assess different sensor technologies worn in parallel during different glycemic ranges and endogenously (e.g., exercise) and exogenously (e.g., insulin) induced glucose swings. These types of studies are required to improve current sensor technologies by means of advanced algorithms and hence to increase the performance of current isCGM and CGM sensors.

## Figures and Tables

**Table 1:** Assessment of intermittently scanned continuous glucose monitoring (isCGM) performance by means of median absolute relative difference (MARD) and median absolute difference (MAD). RCPG = rate of change in plasma glucose; *n* = number of points of comparison; OGTT = oral glucose tolerance test. Values are given as median (interquartile range).

	MARD (%)	MAD (mg/dL)	MAD (mmol/L)	*n*
Overall	8.3 [4.0–14.8]	18 [9–32]	1.0 [0.5–1.8]	242
Pre-OGTT value	6.9 [3.2–10.8]	8 [5–9]	0.4 [0.3–0.9]	34
Hypoglycemia Level 1 (<70 mg/dL [<3.9 mmol/L])	18.8 [15.8–22.0]	17 [13–20]	0.9 [0.7–1.1]	6
Euglycemia (70–180 mg/dL [3.9–10.0 mmol/L])	9.5 [4.3–15.1]	13 [6–22]	0.7 [0.3–1.2]	76
Hyperglycemia level 1 (180–250 mg/dL [10.0–13.9 mmol/L])	9.4 [4.0–17.2]	20 [8–39]	1.1 [0.4–2.2]	65
Hyperglycemia level 2 (>250 mg/dL [>13.9 mmol/L])	7.1 [3.3–11.9]	23 [10–36]	1.3 [0.6–2.0]	95
Low RCPG (0–0.82 mg/dL/min)	6.5 [3.5–10.8]	18 [10–28]	0.9 [0.6–1.5]	51
Moderate RCPG (0.83–1.33 mg/dL/min)	8.4 [3.7–13.2]	17 [9–31]	0.9 [0.5–1.7]	51
High RCPG (1.34–2.33 mg/dL/min)	8.7 [5.0–18.2]	20 [9–36]	1.1 [0.5–2.0]	52
Very high RCPG (>2.33 mg/dL/min)	12.8 [5.1–21.1]	25 [13–45]	1.4 [0.7–2.5]	54

## Data Availability

The data presented in this study are available on request from the corresponding author. The data are not publicly available due to ethical reasons of patient data.
